# Socio-economic and demographic predictors of unmet need for contraception among young women in sub-Saharan Africa: evidence from cross-sectional surveys

**DOI:** 10.1186/s12978-020-01018-2

**Published:** 2020-10-23

**Authors:** Bright Opoku Ahinkorah, Edward Kwabena Ameyaw, Abdul-Aziz Seidu

**Affiliations:** 1 School of Public Health, Faculty of Health, University of Technology Sydney, Sydney, NSW Australia; 2 Department of Population and Health, College of Humanities and Legal Studies, University of Cape Coast, Cape Coast, Ghana; 3 College of Public Health, Medical and Veterinary Sciences, James Cook University, Townsville, QLD Australia

**Keywords:** Socio-economic status, Public health, Unmet need, Contraception, Young women, Reproductive health, Sub-Saharan Africa

## Abstract

**Introduction:**

Globally, sub-Saharan Africa (SSA) bears the highest proportion of women with unmet need for contraception as nearly 25% of women of reproductive age in the sub-region have unmet need for contraception. Unmet need for contraception is predominant among young women. We examined the association between socio-economic and demographic factors and unmet need for contraception among young women in SSA.

**Methods:**

Data for this study obtained from current Demographic and Health Surveys (DHS) conducted between January 1, 2010 and December 31, 2018 in 30 sub-Saharan African countries. The sample size consisted of young women (aged 15–24), who were either married or cohabiting and had complete cases on all the variables of interest (*N* = 59,864). Both bivariate and multivariable binary logistic regression analyses were performed using STATA version 14.0.

**Results:**

The overall prevalence of unmet need for contraception among young women was 26.90% [95% CI: 23.82–29.921], ranging from 11.30% [95% CI: 5.1–17.49] in Zimbabwe to 46.7% [95% CI: 36.92–56.48] in Comoros. Results on socio-economic status and unmet need for contraception showed that young women who had primary [aOR = 1.18; CI = 1.12–1.25, *p* < 0.001] and secondary/higher levels of formal education [aOR = 1.27; CI = 1.20–1.35, *p* < 0.001] had higher odds of unmet need for contraception compared to those with no formal education. With wealth status, young women in the richest wealth quintile had lower odds of unmet need for contraception compared with those in the poorest wealth quintile [aOR = 0.89; CI = 0.81–0.97, *p* < 0.01]. With the demographic factors, the odds of unmet need for contraception was lower among young women aged 20–24 [aOR = 0.74; CI = 0.70–0.77, *p* < 0.001], compared with 15–19 aged young women. Also, young women who were cohabiting had higher odds of unmet need for contraception compared to those who were married [aOR = 1.35; CI = 1.28–1.43, *p* < 0.001].

**Conclusion:**

Our study has demonstrated that unmet need for contraception is relatively high among young women in SSA and this is associated with socio-economic status. Age, marital status, parity, occupation, sex of household head, and access to mass media (newspaper) are also associated with unmet need for contraception. It is therefore, prudent that organisations such as UNICEF and UNFPA and the Bill & Melinda Gates Foundation who have implemented policies and programmes on contraception meant towards reducing unmet need for contraception among women take these factors into consideration when designing interventions in sub-Saharan African countries to address the problem of high unmet need for contraception among young women.

## Introduction

Eighty-nine percent (89.0%) of the global young people reside in low and middle-income countries including sub-Saharan Africa (SSA) [1, 2]. Young people are persons between the ages of 10 and 24 [3]. The proportion of young people in SSA is projected to surge to 605 million by 2050 [4]. Countless factors interplay to determine the health and wellbeing of this burgeoning population, especially females [5]. These factors demand stringent and young women-focused measures that can guarantee the requisite enhancements in their social and reproductive health whilst taking cognisance of the cultural contexts and prevailing institutional structures [5]. Largely, sexual and reproductive health services in SSA have advanced over time, which reflects in more friendly services for young people. Nonetheless, further advancement to commensurate their reproductive health needs is required [6]. This is needful because non-existence of the required reproductive health services intensifies the several risks young women face such as increased chances of contracting sexually transmitted infections as well as unintended pregnancy and its numerous resultant complications [7].

Unmet need for contraception has been a bane for most young women in SSA [8, 9]. The World Health Organisation (WHO) describes women with unmet need for contraception as those who are fecund and sexually active but are not using any method of contraception, and report not wanting any more children or wanting to delay the next birth [10]. Other scholars have also defined women with unmet need for contraception as women who wish to space or limit births but do not use contraceptive methods [11–13]. Ensuing the 2012 London Summit on Family Planning, over 40 states the world over acknowledged that life-saving contraception constitutes a critical aspect of fundamental human rights for women [14].

Globally, SSA bears the highest proportion of women with unmet need for contraception as nearly 25% (i.e. about 47 million) of women of reproductive age in the sub-region fall within this category [15]. This unquestionably accounts for the high fertility and unsafe abortion rates in SSA [16]. In SSA, 3.9% maternal deaths originate from induced abortions. About 19 million unsafe abortions are conducted annually in SSA whilst 25% and 1% of global illegal and legal abortions respectively occur in Africa [17]. The leading cause of unsafe abortion is unmet need for contraception [18]. Abortions dominate among young women in SSA due to cultural, structural and weak health systems that jointly or independently suppress their access to family planning services [19–21]. Easy access, consistent and continuous utilisation of contraception are promising strategies for averting unintended pregnancies, which singularly accounts for nearly all unsafe abortions [22, 23].

As part of efforts to reduce unmet need for contraception in SSA, the United Kingdom of Great Britain and Northern Ireland, UNFPA and the Bill & Melinda Gates Foundation launched the Family Planning 2020 partnership in 2012 with the aim of increasing investment in SSA and other poorest countries in the world, so that 120 million additional women can meet their contraceptive needs by 2020 [24]. Within the sub-region, UNFPA and UNICEF have also implemented programmes aimed at ensuring a steady, reliable supply of quality contraceptives; strengthening national health systems; advocating for policies supportive of family planning; and gathering data to support this work [25].

Considering that most SSA countries are either low or middle-income countries, the socio-economic position at the individual level cannot be discounted in matters relating to unmet need for contraception among young women, who stand the highest risk of unsafe abortion. In Ethiopia, for instance, Dingeta [26] noted that unmet need for contraception declined with women’s social position marked by decision-making capacity. Similar reports have emerged from Nigeria and Cameroon [8, 27]. Poverty lessens women’s prospects of utilising contraception [28]. This may worsen for young women since a greater section of these women may either be in school or still seeking employment opportunities to earn a living, thus an indication of low economic status [29]. In light of the foregoing argument in the literature, socio-economic position of young women in SSA, together with significant demographic characteristics cannot be disentangled from prospects of meeting their contraceptive needs.

Empirical investigation on socio-economic and demographic variations in unmet need for contraception among young women in SSA is limited. Considering the high occurrence of unsafe abortion among young women and high prevalence of unmet need for contraception, we aimed at assessing the association between socio-economic and demographic factors and unmet need for contraception in SSA. Based on the objective of the study, the following hypotheses were considered;

1. There is no statistically significant association between socio-economic status of young women and unmet need for contraception.
2. There is no statistically significant association between the demographic characteristics of young women and unmet need for contraception.

Findings from the study will not only provide socio-economic-induced country specific and sub-regional prevalence of unmet need for contraception but would further direct dialogue and suitable measures required to increase contraceptive coverage among young women in SSA.

## Methods

### Data source

Data for this study was obtained from current Demographic and Health Surveys (DHS) conducted between January 1, 2010 and December 31, 2018 in 30 SSA countries. We focused on 30 countries with recent DHS data (2010–2018), who also had data on unmet need for contraception and other variables considered in this study. The use of countries with recent data was based on the idea that modernization may have an impact on the prevalence of unmet need for contraception in more current surveys compared to older ones. DHS is a nationwide survey undertaken across low and middle-income countries every 5-year period [30]. The survey is representative of each of these countries and targets core maternal and child health indicators such as unmet need for contraception. Women’s files (IR) were used for our study and these files possess the responses of women aged 15–49. In selecting the sample for each survey, stratified dual-stage sampling approach was employed. The first step of this sampling approach involved the selection of clusters (i.e., enumeration areas [EAs]), followed by systematic household sampling within the selected EAs. In this study, the sample size consisted of young women (aged 15–24), who were either married or cohabiting and had complete cases on all the variables of interest (*N* = 59,864). The study followed the ‘Strengthening the Reporting of Observational Studies in Epidemiology’ (STROBE) statement in conducting this study and writing the manuscript (see Additional file: Table 1). Detailed information on the survey country, year of survey and sample size for each country has been provided in Table 1.

**Table 1:** Description of the study sample

Country	Survey year	Sample (*N*)
Angola	2015–16	2237
Benin	2017–18	2531
Burkina Faso	2010	3482
Burundi	2016–17	1641
Cameroon	2011	2709
Chad	2014–15	3667
Comoros	2012	677
Congo	2011–12	1479
Congo DR	2013–14	3170
Côte d'Ivoire	2011–12	1477
Ethiopia	2016	2399
Gabon	2012	1047
Gambia	2013	1811
Ghana	2014	761
Guinea	2018	1752
Kenya	2014	1783
Lesotho	2014	259
Liberia	2013	1232
Malawi	2015–16	4679
Mali	2018	2325
Mozambique	2015	1286
Namibia	2013	486
Nigeria	2018	3197
Rwanda	2014–15	911
Senegal	2010–11	2945
Sierra Leone	2013	2163
Togo	2013–14	1120
Uganda	2016	3286
Zambia	2013–14	2182
Zimbabwe	2015	1413
Total	–	59,864

### Study variables

#### Dependent variable

The outcome variable for this study is dichotomized as unmet need (yes/no) which was generated from a constructed variable in the DHS. It is the sum of unmet need for spacing and limiting and young women who were married, fecund and/or sexually active have unmet needs if they don’t want any more children or want to delay their next birth for at least two years but not using contraception. Pregnant or amenorrheic young women with unwanted or mistimed pregnancies or births were also considered to have unmet if they were not using contraception at the time they conceived [31, 32].

#### Explanatory variables

The main explanatory variable for this study was socio-economic status. This variable consists of two variables (wealth status and level of education) which have been considered as key indicators for measuring socio-economic status [33–35]. In computing wealth in the standard DHS, data on household ownership of selected assets such as bicycle, materials used for house construction, television, type of water access and sanitation facilities were used. Wealth status was then created from these assets through Principal Component Analysis (PCA) by placing households on a continuous measure of relative wealth after which households were grouped into five wealth quintiles namely poorest, poorer, middle, richer and richest [36]. Maternal level of education, on the other hand, is a standardized variable that categorises education into no education, primary, secondary, and higher [36]. We maintained the original categorization and coding of these two independent variables, (i. e. wealth status and level of education).

Apart from these variables, survey country, age, sex of household head, marital status, religion, place of residence, occupation, frequency of reading newspaper/magazine, frequency of listening to radio, frequency of watching television, and parity were considered as covariates. Except survey country, which was selected a priori, the rest were selected based on their availability in the dataset and their significant associations with unmet need for contraception in previous studies [31, 32, 37–39]. In order to make all these variables conceptually meaningful and suitable for the analysis, some of them were recoded. Age was coded into “15–19” and “20–24”. Religion was recoded as “Christianity”, “Islam”, “Traditional religion” and “No religion”. Parity was categorised as “Zero birth”, “One birth”, “Two births”, and “Three or more births”. Frequency of reading newspaper/magazine, listening to radio and watching television were grouped as “Not at all”,“Less than once a week”, and “At least once a week”. Lastly, employment status was coded as “ Unemployed” and “Employed”.

### Statistical analysis

**Fig. 1:**
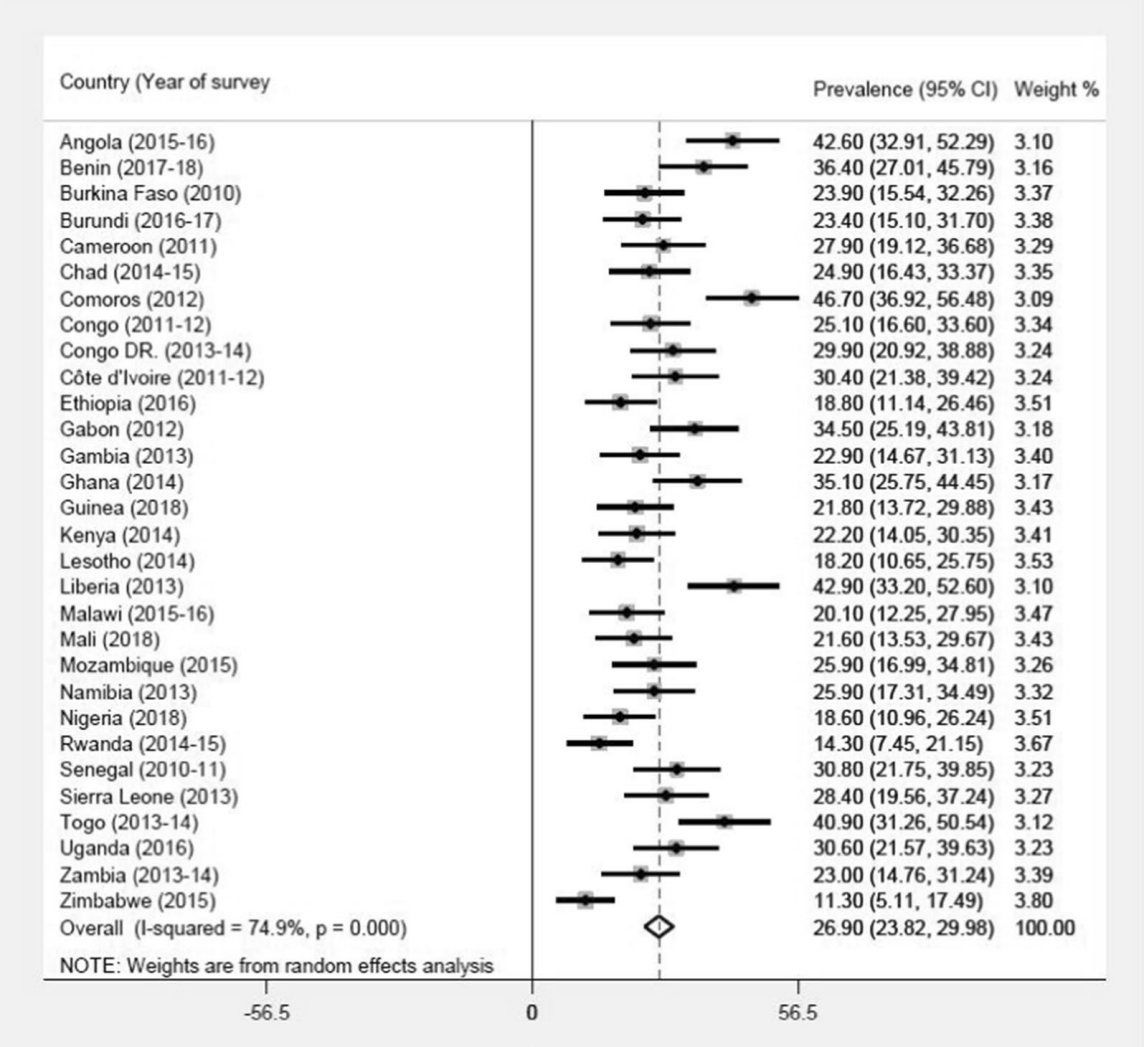
Proportion of young women in sub-Saharan Africa with unmet need for contraception

A three-step analytical approach was followed in analysing the data to produce the results for the study. The first step involved the computation of unmet need for contraception among young women in SSA. This was done by generating a forest plot using the syntax “metan” command in STATA version 14.0 (StataCorp, College Station, TX, USA). The forest plot showed the prevalence of unmet need for contraception in individual countries and its corresponding weight, as well as the pooled prevalence of unmet need for contraception in all the countries and their associated 95% confidence intervals (CI). Before this, a test of heterogeneity of the DHS data was done which showed a high level of inconsistency (*I*^2^ > 50%) and this warranted the use of a random effect model in the meta-analysis (see Fig. 1).

Secondly, we appended the datasets and calculated the prevalence of unmet need for contraception across the socio-demographic characteristics of the respondents with their corresponding significance levels and chi square values [*χ*^2^] (see Table 2). To check for correlation among the explanatory variables, a test for multicollinearity was done using the variance inflation factor (VIF) and the results showed no evidence of high collinearity among the explanatory variables (Mean VIF = 1.29, Maximum VIF = 1.62, and Minimum VIF = 1.02**)**. We applied sample weight (v005/1,000,000) to correct for over and under sampling while the SVY command was used to account for the complex survey design and generalizability of the findings.

**Table 2:** Socio-economic status and unmet need for contraception among young women in SSA

Variables	Weighted *N*	Weighted %	Unmet need for contraception	
			Weighted *N*	Weighted %
*Educational level [χ*^*2*^* = 24.9, p < 0.001]*				
No education	20,027	33.5	5246	25.3
Primary	21,735	36.3	5899	27.2
Secondary/higher	18,102	30.2	4744	27.2
*Wealth status [χ*^*2*^* = 36.7, p < 0.001]*				
Poorest	12,904	21.6	3890	26.6
Poorer	13,655	22.8	3684	27.0
Middle	12,314	20.6	3321	27.6
Richer	11,735	19.6	2926	26.8
Richest	9,255	15.5	2068	24.0
*Age [χ*^*2*^* = 5.2, p < 0.05]*				
15–19	16,755	28.0	4592	27.2
20–24	43,109	72.0	11,297	26.3
*Sex of household head [χ*^*2*^* = 101.0, p < 0.001]*				
Male	50,679	84.7	12,848	25.6
Female	9,185	15.3	3041	31.6
*Place of residence [χ*^*2*^* = 35.4, p < 0.001]*				
Urban	18,508	30.9	5079	28.2
Rural	41,356	69.1	10,810	25.8
*Marital status [χ*^*2*^* = 454.8, p < 0.001]*				
Married	44,407	74.2	10,791	24.3
Cohabiting	15,457	25.8	5098	26.5
*Employment status [χ*^*2*^* = 39.3, p < 0.001]*				
Unemployed	22,902	38.3	6640	27.9
Employed	36,962	61.7	9249	25.6
*Religion [χ*^*2*^* = 38.0, p < 0.001]*				
Christianity	34,303	57.3	9081	27.0
Islam	22,748	38.0	5959	25.4
Traditional religion	1,431	2.4	409	27.8
No religion	1,384	2.3	440	31.5
*Frequency of reading newspaper/magazine [χ*^*2*^* = 12.9, p < 0.01]*				
Not at all	51,107	85.4	13,811	26.8
Less than once a week	5,070	8.5	1221	24.5
At least once a weak	3,687	6.2	857	25.8
*Frequency of listening to radio [χ*^*2*^* = 5.1, p > 0.05]*				
Not at all	21,003	45.1	7331	26.7
Less than once a week	11,012	18.4	2977	27.2
At least once a weak	21,849	36.5	5581	26.5
*Frequency of watching television [χ*^*2*^* = 22.8, p < 0.001]*				
Not at all	37,744	63.1	10,012	25.9
Less than once a week	6,800	11.4	1830	26.9
At least once a weak	15,319	25.6	4047	28.0
*Parity [χ*^*2*^* = 731.7, p < 0.001]*				
Zero births	10,818	18.1	1798	16.7
One birth	22,969	38.4	6162	27.1
Two births	16,381	27.4	4716	28.8
Three or more births	9,697	16.2	3213	32.0

Finally, binary logistic regression models were fitted in a hierarchical order using the explanatory variables which were significantly associated with unmet need for contraception (*p* < 0.05) from the chi-square analysis. The first model was an incomplete multivariable model, which consisted of socio-economic status [measured by wealth status and level of education], and the outcome variable [unmet need for contraception]. Model II, on the other hand, looked at all the explanatory variables and the outcome variable (see Table 3). Finally, model III was a complete model that looked at the influence of all the explanatory variables on the outcome variable, while controlling for survey countries. Results for the regression analysis have been presented as crude odds ratios (cOR) and adjusted odds ratios (aOR) with their corresponding 95% confidence intervals (CI) signifying precision and significance of the reported OR values. Statistical significance was declared at *p* < 0.05. Test for model fit was done using the Hosmer–Lemeshow test and reported as Pseudo *R*^2^.

**Table 3:** Binary logistic regression on socio-economic status and unmet need for contraception among young women in SSA

Variables	Model I cOR[95%CI]	Model II AOR[95%CI]	Model III AOR[95%CI]
*Educational level*			
No education	Ref	Ref	Ref
Primary	1.11^***^[1.06–1.16]	1.07^*^[1.01–1.12]	1.18^***^[1.12–1.25]
Secondary/higher	1.15^***^[1.10–1.21]	1.13^***^[1.07–1.20]	1.27^***^[1.20–1.35]
*Wealth status*			
Poorest	Ref	Ref	Ref
Poorer	1.01[0.96–1.06]	1.02[0.97–1.08]	1.01[0.95–1.06]
Middle	1.03[0.97–1.09]	1.03[0.97–1.09]	1.02[0.96–1.08]
Richer	0.98[0.93–1.04]	0.97[0.91–1.03]	1.01[0.95–1.08]
Richest	0.83^***^[0.78–0.89]	0.82^***^[0.76–0.88]	0.89^**^[0.81–0.97]
*Age*			
15–19		Ref	Ref
20–24		0.74^***^[0.70–0.77]	0.74^***^[0.70–0.77]
*Sex of household head*			
Male		Ref	Ref
Female		1.31^***^[1.25–1.38]	1.30^***^[1.24–1.37]
*Place of residence*			
Urban		Ref	Ref
Rural		0.88^***^[0.83–0.92]	0.95[0.90–1.00]
*Marital status*			
Married		Ref	Ref
Cohabiting		1.55^***^[1.49–1.62]	1.35^***^[1.28–1.43]
*Employment status*			
Unemployed		Ref	Ref
Employed		0.88^***^[0.85–0.91]	0.87^***^[0.83–0.90]
*Religion*			
Christianity		Ref	Ref
Islam		1.09^***^[1.04–1.14]	0.98[0.92–1.04]
Traditional religion		0.99[0.88–1.12]	0.91[0.80–1.04]
No religion		1.16^*^[1.03–1.31]	1.04[0.92–1.18]
*Frequency of reading newspaper/magazine*			
Not at all		Ref	Ref
Less than once a week		0.87^***^[0.81–0.94]	0.93[0.87–1.01]
At least once a week		0.89^*^[0.82–0.97]	0.904^*^[0.83–0.99]
*Frequency of watching television*			
Not at all		Ref	Ref
Less than once a week		1.09^**^[1.02–1.16]	1.07[1.00–1.14]
At least once a week		1.09^**^[1.04–1.15]	1.01[0.95–1.07]
*Parity*			
Zero births		Ref	Ref
One birth		2.03^***^[1.91–2.15]	2.08^***^[1.96–2.21]
Two births		2.43^***^[2.28–2.59]	2.49^***^[2.33–2.66]
Three or more births		2.97^***^[2.77–3.19]	2.99^***^[2.78–3.21]
*Survey country*			
Angola			4.51^***^[3.71–5.49]
Burkina Faso			3.39^***^[2.79–4.11]
Benin			5.39^***^[4.45–6.53]
Burundi			2.68^***^[2.18–3.31]
Congo DR			3.48^***^[2.89–4.20]
Congo			2.18^***^[1.75–2.71]
Côte d'Ivoire			3.84^***^[3.11–4.73]
Cameroon			3.07^***^[2.54–3.71]
Ethiopia			2.29^***^[1.87–2.80]
Gabon			3.14^***^[2.52–3.92]
Ghana			4.42^***^[3.52–5.56]
Gambia			2.90^***^[2.36–3.58]
Guinea			2.73^***^[2.21–3.37]
Kenya			2.42^***^[1.98–2.97]
Comoros			7.88^***^[6.21–9.99]
Liberia			5.03^***^[4.08–6.19]
Lesotho			2.29^***^[1.59–3.30]
Mali			2.60^***^[2.12–3.19]
Malawi			2.23^***^[1.86–2.69]
Mozambique			2.44^***^[1.98–3.02]
Nigeria			2.18^***^[1.79–2.65]
Namibia			2.26^***^[1.71–3.00]
Rwanda			1.39^*^[1.08–1.80]
Sierra Leone			3.73^***^[3.06–4.56]
Senegal			4.42^***^[3.63–5.37]
Chad			2.90^***^[2.40–3.51]
Togo			6.30^***^[5.10–7.77]
Uganda			3.28^***^[2.72–3.96]
Zambia			2.43^***^[2.00–2.96]
Zimbabwe			Ref
*N*	59,864		59,864
pseudo *R*^2^	0.001	0.025	0.043

Exponentiated coefficients; 95% confidence intervals in brackets

*Ref* reference, *CI* confidence intervals, *COR* crude odds ratio, *AOR* adjusted odds ratio

^*^p < 0.05, ^**^p < 0.01, ^***^p < 0.001

### Ethical approval

The DHS surveys obtain ethical clearance from the Ethics Committee of ORC Macro Inc. as well as Ethics Boards of partner organisations of the various countries such as the Ministries of Health. During each of the surveys, either written or verbal consent was provided by the women. Since the data was not collected by the authors of this manuscript, permission was sought from MEASURE DHS website and access to the data was provided after our intent for the request was assessed and approved on 3rd April, 2019. Data is available on https://dhsprogram.com/data/available-datasets.cfm.

## Results

The prevalence of unmet need for contraception among young women in each of the 30 SSA countries included in the study are presented in Fig. 1. The overall prevalence of unmet need for contraception was 26.90% [95% CI: 23.82–29.921], ranging from 11.30% [95% CI:5.1–17.49] in Zimbabwe to 46.7% [95% CI: 36.92–56.48] in Comoros.

Table 2 provides a summary of the socio-demographic characteristics, the proportion of unmet need and their association of significance. Young women with primary and secondary/higher level of education had greater proportion of unmet need for contraception [27.2%]. Unmet need for contraception was high among young women in the middle wealth quintile [27.6%] compared to those in the richest wealth quintile [24.0%]. In terms of age, young women aged 15–19 had the highest proportion of unmet need for contraception compared to those aged 20–24. Young women in female-headed households (31.6%), those in urban areas (28.2%), those cohabiting (26.5%), those not working (27.9%), those with no religion (31.5%) and those with three or more births (32%) had the highest prevalence of unmet need for contraception. With media exposure, those who do not read newspaper/magazine at all (26.7%), those who watch television at least once a week (28%) and those who listen to radio less than once a week had the highest prevalence of unmet need for contraception. All the explanatory variables showed statistically significant associations with unmet need for contraception at 95% confidence interval, except frequency of listening to radio (see Table 2).

### Binary logistic regression on socio-economic status and unmet need for contraception among young women in SSA

Results in Table 3 on socio-economic status and unmet need for contraception showed that young women who had primary [aOR = 1.18; CI = 1.12–1.25, *p* < 0.001] and secondary/higher levels of formal education [aOR = 1.27; CI = 1.20–1.35, *p* < 0.001] had higher odds of unmet need for contraception compared to those with no formal education. With wealth status, young women in the richest wealth quintile had lower odds of unmet need for contraception compared with those in the poorest wealth quintile [aOR = 0.89; CI = 0.81–0.97, *p* < 0.01]. The odds of unmet need for contraception was low among young women aged 20–24 [aOR = 0.74; CI = 0.70–0.77, *p* < 0.001] compared with 15–19 aged young women. The results further showed that young women in female headed households [aOR = 1.30; CI = 1.24–1.37, *p* < 0.001] had higher odds of unmet need for contraception compared with those in male headed households.

Also, young women who were cohabiting had higher odds of unmet need for contraception compared to those who were married [aOR = 1.35; CI = 1.28–1.43, *p* < 0.001]. The odds of unmet need for contraception was low among young women who were employed [aOR = 0.87; CI = 0.83–0.90, *p* < 0.001] compared to those who were unemployed. Furthermore, young women who read newspaper/magazine at least once a week had lower odds of unmet need for contraception compared to those who did not read newspaper at all [aOR = 0.904; CI = 0.83–0.99, *p* < 0.05]. Finally, the odds of unmet need for contraception increased with parity. Specifically, those with three or more births [aOR = 2.99; CI = 2.78–3.21, *p* < 0.001] had the highest odds of unmet need for contraception compared with nulliparous young women. Compared to Zimbabwe, all the countries had higher odds of unmet need for contraception. However, young women in Comoros had the highest odds of unmet need for contraception [aOR = 7.88; CI = 6.21–9.99, *p* < 0.001] (see Table 3).

## Discussion

In this study, we assessed the association between demographic and socio-economic status of young women and unmet need for contraception in 30 countries in SSA. The study showed statistically significant association between socio-economic status (wealth status and education) and unmet need for contraception, while controlling for survey country, marital status, parity, occupation, sex of household head, age of respondent, access to mass media (newspaper). We found that the prevalence of unmet need for contraception among young women was 26.9%. The prevalence recorded in this study is comparable to previous studies in Libya [40], Gambia [7] and Norway [41]. However, the prevalence in this study is lower than what was found in other parts of SSA such as 34.6–44% in Ethiopia [26, 42], 32.4% in Burundi [43], 31.1% in Cameroon [27] and 30% in Ghana [23]. The prevalence is also higher than the prevalence in other studies such as 16.2–17.4% in Ethiopia [17, 39], 17.4% in Iran [44], 10–18% in Nigeria [45, 46], 17% in Indonesia [47], 11.5% in Mexico [40] and 10.5% in Latin America [48]. The probable reason for the disparities in findings could be due to the differences in location, study population and time they were conducted.

It was found that, young women in all the countries had higher odds of unmet need for contraception compared with Zimbabwe. Nonetheless, Comoros had the highest proportion of unmet need for contraception. The regression analysis confirmed this by showing that young women in Comoros had about seven times higher odds of unmet need for contraception compared with those in Zimbabwe. The high unmet need for contraception among young women in Comoros is not surprising in this current study. Previous evidence in Comoros indicate that there has been a stagnation in the progress of contraceptives prevalence in Comoros among women in their reproductive age in general [49]. The 2012 Demographic and Health Survey of Comoros also revealed that as high as 80% of women in their reproductive age are not using contraceptives [50]. A study by Rai [49] also indicated that, a greater proportion of women in Comoros do not even intend to use contraceptives in the near future. This finding, therefore, suggest the need for more empirical studies, both qualitative and quantitative to unearth the various factors accounting for low contraceptive usage and the high unmet need for contraception in Comoros since our study and others [49, 50] suggest barriers in both access and utilisation of contraceptives in the country. In addition, there is the need for the government to ensure the provision of contraceptives and also adequately engage in behaviour change communication interventions to offset possible barriers to contraceptive uptake in the country [49]. It is also prudent for various countries in SSA to institute pragmatic measures to make contraceptives more accessible to young women and educate them on the need to use contraceptives. This can go a long way to reduce unintended pregnancies and reduce maternal mortality.

The study also showed that socio-economic status is associated with unmet need for contraception. Specifically, with wealth status, the study indicated that young women in the richest wealth quintile had lower odds of unmet need for contraception compared with the poorest. This association has been observed in previous studies in other parts of the world such as Libya [40], Ghana [23], Pakistan [51] and Ethiopia [39]. The probable explanation is that younger women from richer/wealthier households can have better access to modern contraceptives as compared to poorer households [51] since they can foot both the direct and indirect cost associated with contraceptive uptake. Young women in this category are also more likely to be enlightened to understand information relating to contraceptive uptake. With level of education, the study revealed that young women with secondary/higher level of education had higher odds of unmet need for contraception compared with those with no formal education. This is similar to what Solanke et al. [45] found in Nigeria and Guure et al. [23] in Ghana. We admit just as Solanke et al. [45] admitted in their study that this finding seem to be counter intuitive. The possible reason accounting for this finding might be that young women who have attained higher level of education, have higher likelihood of postponing marriage or childbearing. However, our findings are different from what has been observed in Mexico [43], Nigeria [52, 53], Kenya [54], Pakistan [51] and Ghana [55] which showed inverse relationship between educational level and unmet need for contraception. This finding warrant further interrogation with a qualitative study to understand the nuances.

In terms of age of young women and unmet need for contraception, we found that those aged 20–24 had lower odds of unmet need for contraception compared to those aged 15–19. This confirms previous studies in Mexico [40], Ethiopia [39, 56] and a SSA-based study [57] which showed that unmet need was highest among adolescents. Various pathways could explain this observation. Adolescents might have various barriers in terms of access to contraceptives ranging from stigma, cost, geographical, shyness, and inadequate information on contraception [39].

Marital status also showed a statistically significant association with unmet need for contraception with cohabiting young women having higher odds of unmet need for contraception. This is consistent with previous studies [40, 58] in SSA. This is nonetheless, contrary to a previous study in Hungary [59]. Some studies have shown that opposition from partners [60, 61] is the reason why cohabiting women have more unmet need for contraception. Relatedly, young women in female-headed households had higher odds of unmet need. This finding warrant further study to get deeper explanation. Young women who are working had lower odds of unmet need. This finding is in line with previous studies [23, 48, 51]. This might be due to the decision making power and the ability to afford both direct and indirect costs associated with contraceptive uptake compared with young women who are not working.

Access to mass media (newspaper) also showed statistically significant association with unmet need. Those who are exposed to newspaper had lower odds of unmet needs. The probable explanation is that young women might get some education from this source in relation to the availability and usefulness of different methods of contraception. Thus, an effective media campaign can be useful to reduce unmet need for family planning among young women in SSA [51]. Young women with three or more children had highest unmet need for contraception which corroborates previous evidence in Nigeria [45], Pakistan [51] and Ethiopia [4, 62]. This result might be an indication that most of the pregnancies and births by young women in SSA are unplanned as proposed by Ameyaw et al. [63] and Tadele, Abebaw, and Ali [39]. It is therefore, crucial to institute measures to help increase contraceptives usage since unintended pregnancies and more births are associated with adverse outcomes including death especially among young women and adolescents [45, 64].

### Strength and limitations

The strength of this study is the use of nationally representative datasets to measure the effect of socio-economic status and demographic factors on unmet need for contraception among young women in SSA. The large sample size and the adoption of well laid procedures such as training of experienced field enumerators and the use of validated instruments strengthen the validity of findings from the dataset. However, since the data on unmet need for contraception, socio-economic status and other covariates were collected at the same time, it is impossible to establish causality. There is also the possibility that young women will provide social desirable responses and may also find it challenging to recollect previous events which could impose recall bias on the study.

## Conclusion

In conclusion, our study has demonstrated that unmet need for contraception is relatively high among young women in SSA and this is associated with their socio-economic status. It was also found that age, marital status, parity, occupation, sex of household head, and access to mass media (newspaper) are associated with unmet need for contraception among young women in SSA. It is therefore, prudent that organisations such as UNICEF and UNFPA and the Bill & Melinda Gates Foundation who have implemented policies and programmes on contraception meant towards reducing unmet need for contraception among women take these factors into consideration when designing interventions in SSA countries to address the problem of high unmet need for contraception among young women.

## Supplementary information

**Additional file 1: Table 1.** STROBE 2007 (v4) Statement—Checklist of items that should be included in reports of cross-sectional studies

## Data Availability

Data for this study were sourced from Demographic and Health surveys (DHS) and available here: https://dhsprogram.com/data/available-datasets.cfm

## Abbreviations

SDG: Sustainable development goals, sub-Saharan Africa
DHS: Demographic and health surveys
COR: Crude odds ratios
AOR: Adjusted odds ratio
CI: Confidence interval
WHO: World Health Organization
ICF: Inner city fund
USAID: United States Agency for International Development
UNDP: United Nations Development Programme
PCA: Principal component analysis
